# Estimating the relative contributions of maternal genetic, paternal genetic and intrauterine factors to offspring birth weight and head circumference

**DOI:** 10.1016/j.earlhumdev.2010.05.021

**Published:** 2010-07

**Authors:** Frances Rice, Anita Thapar

**Affiliations:** a Division of Psychology and Language Sciences, University College London, 26 Bedford Way, London, WC1H 0AP, United Kingdom; b Department of Psychological Medicine and Neurology, MRC Centre in Neuropsychiatric Genetics and Genomics, Cardiff University School of Medicine, Heath Park, Cardiff, CF14 4XN, United Kingdom

**Keywords:** Birth weight, IVF, Prenatal, Genetic, Intrauterine, Growth

## Abstract

**Background:**

Genetic factors and the prenatal environment contribute to birth weight. However, very few types of study design can disentangle their relative contribution.

**Aims:**

To examine maternal genetic and intrauterine contributions to offspring birth weight and head circumference. To compare the contribution of maternal and paternal genetic effects.

**Study design:**

Mothers and fathers were either genetically related or unrelated to their offspring who had been conceived by in vitro fertilization.

**Subjects:**

423 singleton full term offspring, of whom 262 were conceived via homologous IVF (both parents related), 66 via sperm donation (mother only related) and 95 via egg donation (father only related).

**Measures:**

Maternal weight at antenatal booking, current weight and maternal height. Paternal current weight and height were all predictors. Infant birth weight and head circumference were outcomes.

**Results:**

Genetic relatedness was the main contributing factor between measures of parental weight and offspring birth weight as correlations were only significant when the parent was related to the child. However, there was a contribution of the intrauterine environment to the association between maternal height and both infant birth weight and infant head circumference as these were significant even when mothers were unrelated to their child.

**Conclusions:**

Both maternal and paternal genes made contributions to infant birth weight. Maternal height appeared to index a contribution of the intrauterine environment to infant growth and gestational age. Results suggested a possible biological interaction between the intrauterine environment and maternal inherited characteristics which suppresses the influence of paternal genes.

## 1 Introduction

Population-based cohort studies and follow-up studies of low birth weight infants have shown that reduced infant birth weight is associated with a wide range of health, cognitive, behavioural and functional difficulties in childhood and adulthood. These adverse sequelae are thought to arise from early programming effects [1–14] Interpretations of research reports of association with low birth weight and offspring outcome are often based on the assumption that birth weight is a reliable index of the intrauterine environment. However, it is also known that genetic factors play a role in the aetiology of infant birth weight [15–17]. Thus, associations between low birth weight and poor offspring outcomes could be at least partly attributable to a shared inherited aetiology rather than to environmentally mediated programming effects [17–19]. Studies examining the familial correlation for birth weight in parent–offspring pedigrees have illustrated that maternally provided genetic factors influence infant birth weight [20].

Differential maternal and paternal genetic contributions to infant birth weight may also be important. To date, the evidence suggests that the effect of maternally provided genes on infant birth weight is greater than that of paternally provided genes [21] or that there are paternal genetic contributions primarily to skeletal growth rather than to overall growth [22]. However, only a few studies have compared the relative contribution of maternally and paternally provided genetic factors to infant birth weight. A difficulty in examining the relative contribution of intrauterine factors with maternal and paternal genetic contributions to infant birth weight is that few types of study design are able to disentangle these different influences. The existing literature on the genetic and environmental aetiology of birth weight is also inconsistent: the results of one study suggest that intrauterine factors are the most important influence on birth weight with a negligible contribution of maternal genetic factors [23]. In contrast, other studies report a substantial influence of maternally provided genetic influences and negligible paternal genetic influences or ignore paternal genetic influences altogether [20,24]. Finally, other studies show evidence of paternal genetic contributions to some but not all aspects on infant growth [22]. To date, no study has simultaneously estimated paternal and maternal genetic contributions as well as the contribution of the intrauterine environment. Given the influence that low birth weight has on a wide range of health conditions, an improved understanding of the causes of variation in birth weight would be a useful contribution to the literature. It could also help to provide opportunities for prevention and intervention during the perinatal period.

Here we used a novel approach to examine the contributions of maternally provided genetic influences, intrauterine influences and paternally provided genetic influences on infant birth weight by examining children who differ in their genetic relatedness to mother and father because of assisted reproductive technology (homologous IVF, IVF with sperm donation and IVF with egg donation).

## 2 Method

### 2.1 Study design

#### 2.1.1 Identifying an inherited effect on size at birth

The contribution of maternal genes to infant birth weight can be identified by comparing mother–offspring associations in the two groups where mother shares genes with their offspring (homologous and sperm donation groups) to the correlation in the egg donation group (See Table 1).

#### 2.1.2 Identifying a prenatal effect on size at birth

The clearest indication of intrauterine factors is provided by the magnitude of mother–offspring correlation in the egg donation group since significant mother–offspring correlation in this group can only be due to intrauterine factors as mother and child do not share genes. Mother–offspring and father–offspring comparisons can also be informative as higher mother–offspring associations are consistent with prenatal effects (although see following section).

#### 2.1.3 Identifying parent of origin inherited influences

Within conception group comparisons of the mother–offspring and father–offspring correlations are informative for identifying the relative contributions of maternal and paternal genetic contributions to infant size at birth. Within the homologous group, mother–offspring and father–offspring correlations should be similar to the extent that maternal and paternal genetic contributions to infant birth weight are similar. Within the sperm donation group, if genes contribute to birth weight the mother–offspring correlation should be larger than the father–offspring correlation. Within the egg donation group, if genes contribute the father–offspring correlation should be larger than the mother–offspring correlation.

##### 2.1.3.1 Sample

Families who had a live birth between 1994 and 2002 (children aged 4 to 10 years) following successful IVF treatment from any of the three conception groups described were recruited from 19 UK clinics and 1 USA clinic. Gamete donors were unrelated to either parent. All initial contact was made through clinic staff. Data were collected through postal questionnaires and, where consent was provided, review of antenatal records. The majority of women agreed for their antenatal records to be reviewed (77%) although we did not request antenatal notes for participants recruited from the USA clinic. Wales Multi Centre Research Ethics Committee reviewed and approved the study. Mothers and fathers were sent a postal questionnaire that included scales to assess various aspects of the pregnancy and demographic characteristics. Mothers reported on pregnancy and obstetric complications using an adapted version of the Lewis and Murray scale [28].

#### 2.1.4 Selection of singletons born at term

**Fig. 1:**
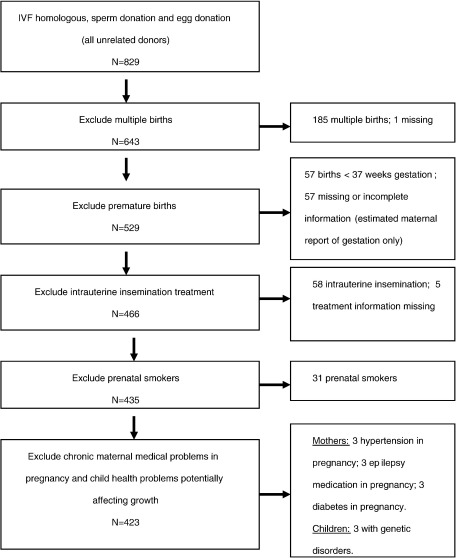
Selection of final sample of singletons born at term for analysis.

This report focuses on singleton births born at term (>=37 weeks gestation) from the three conception groups described given the strong influences of prematurity and multiple birth on birth weight [25]. Moreover, the processes underlying the aetiology of birth weight in infants born at term and those born prematurely may differ [29] and we sought to examine genetic and intrauterine influences on normal variation in birth weight. Fig. 1 outlines the selection of the sample of singletons born at term from the full sample. The multiple birth rate in the sample as a whole was 24% which is in line with other samples of children born following assisted reproductive technologies [30]. We included children conceived following IVF (in vitro fertilization) (*N* = 384), ICSI (intra cytoplasmic sperm injection) (*N* = 76) and GIFT (gamete intrafallopian transfer) (*N* = 6) and excluded children born following intrauterine insemination (*N* = 58). Treatment details were missing for 5 cases. We also excluded prenatal smokers (*N* = 31) given the known effect of prenatal smoking on offspring birth weight [25,26]. In addition, mothers with chronic physical health problems during pregnancy that could have affected infant growth (*N* = 8) were excluded. Specifically, we excluded two women with diabetes, three women taking anti-epileptic medication throughout pregnancy and three women taking medication for hypertension throughout pregnancy. Finally, children with serious medical conditions that could have influenced prenatal growth (*N* = 3) were also excluded. These were three children with genetic syndromes (Down's syndrome, lissencephaly and anhidrotic ectodermal dysplasia). Following these exclusion criteria, questionnaire data from at least one parent was available for 423 families; 262 homologous IVF (parents own gametes used) (259 mother questionnaires; 187 father questionnaires; 184 both parent questionnaires), 66 IVF with sperm donation (66 mother questionnaires; 47 father questionnaires; 46 both parent questionnaires), and 95 IVF with oocyte donation (95 mother questionnaires; 64 father questionnaires; 64 both parent questionnaires). Data from antenatal records were available for 277 (65%) of these mothers (176 homologous; 46 sperm donation; 55 egg donation).

## 3 Measures

### 3.1 Offspring outcomes

#### 3.1.1 Birth weight

In accordance with our previous work [31], agreement between maternal reports and antenatal records for birth weight was nearly perfect in this sample (*r* = .972, *p* = .001, *n* = 277; mean difference = − 15.82, sd = 112.69). We therefore used maternal reports which allowed us to use data from the full sample as opposed to the subsample for whom antenatal records were available. Agreement between maternal and antenatal reports for low birth weight <=2500 g was also very good (Kappa = .818, *p* = .001).

#### 3.1.2 Birth weight corrected for gestational age

Since birth weight is both a function of foetal growth and of gestational age we additionally calculated birth weight adjusted for weeks gestation. The unstandardised residuals were saved from a regression where gestation in weeks was the predictor and birth weight was the outcome.

#### 3.1.3 Head circumference

This measure was available from antenatal records.

### 3.2 Measures of parental stature

#### 3.2.1 Parental height

Mothers and fathers reported on their height. Responses were converted to metres. Maternal report of height in the questionnaire and information from antenatal records showed good agreement (mean difference = .0075, sd = .026) indicating that on average mothers reported their height within 1 cm of their measured height.

#### 3.2.2 Maternal pregnancy weight

Maternal weight at antenatal booking was available for 215 mothers (mean week of pregnancy weighed = 12.57) from antenatal records.

#### 3.2.3 Maternal and paternal current weight

Although on average, children were aged 6 years at the time these data were collected, we also included maternal and paternal reports of current weight for two reasons. First, to examine whether the pattern of results obtained for maternal pregnancy weight could be replicated using this less stringent measurement variable of maternal weight. Second, to allow a direct comparison of the relative contributions of maternal and paternal weight to infant size. Mothers and fathers reported on their current weight. All responses were converted to kilograms. Current maternal weight was very similar to weight at antenatal booking (mean difference = −.07, sd = 6.16). Thus, on average women currently weighed .07kg more than they weighed in pregnancy.

### 3.3 Potential influences on infant size

Mothers reported on their child's gender, whether the child was their first born child and maternal and paternal age when their child was born. Mothers reported their annual family income on a six point scale ranging from <£10,000 to >£60,000 and completed an adapted version of the Lewis and Murray antenatal complications scale [28]. This scale has shown good to excellent agreement with information from antenatal records for most variables [31].

### 3.4 Statistics

We first analysed data from the whole sample and used correlations, *t*-tests or ANOVA tests as appropriate to examine the influence of a number of variables on infant birth weight. We also compared the frequency of the potential confounding factors described above between the three conception groups. Next, we examined the unadjusted correlations between maternal and paternal height and weight with infant birth weight separately in the three conception groups (homologous, sperm donation, and egg donation). We used linear regression to adjust for potential confounders in the association between parental height and weight with infant birth weight. Finally, we calculated maternal–offspring and paternal–offspring correlations using birth weight adjusted for gestational age as an outcome variable.

## 4 Results

### 4.1 Descriptives

Descriptive characteristics of the full sample have been described in detail elsewhere [18,19,32]. In this subsample of singleton births born at term, there were 222 female and 201 male offspring and the gender composition did not differ between the three conception groups (*χ*^2^ = 3.038, df = 2, *p* = .219). The majority of mothers and fathers described their ethnic group as white British (96% of mothers and fathers). 81% of mothers in the sample were primiparous and there was no difference in the proportion of primiparous women in the different conception groups (*χ*^2^ = 0.006, df = 2, *p* = .977). The mean birth weight for the sample was 3385 grams (sd = 502) which is consistent with UK population norms for singletons of White British ethnicity [33]. On average, offspring were born at 39.57 weeks gestation (sd = 1.40). The average family income for the sample was between £30,000 and £40,000 and this did not differ between the conception groups (*F* (2,403) = 2.131, *p* = .12).

### 4.2 Birth weight

There was no significant difference between the three conception groups (homologous, sperm donation, egg donation) on infant birth weight (F = 0.821 (2, 420), *p* = .441). Parity (primiparous and multiparous) did not significantly influence birth weight (*t* = .194 df = 402, *p* = .846). There were no significant differences between the three conception groups in terms of maternal reported antenatal complications with the exception that women in the egg donation group reported higher levels of oedema/high blood pressure in pregnancy (*χ*^2^ = 16.62, df = 2, *p* = .001) and there was a trend for this to be associated with lower birth weight (*t* = 1.838, df = 418, *p* = .067). Maternal age and paternal age at child birth were also not associated with infant birth weight (*r* = .007, *p* = .885 and *r* = −.031, *p* = .521) respectively. However, child gender and family income did influence birth weight: males were significantly heavier at birth than females (*t* = 3.121, df = 421, *p* = .002; mean male = 3464 g; mean female = 3313 g) and families with an annual income under £10,000 per year (*n* = 14) had lower birth weight babies than the remainder of the sample (*t* = 2.285, df = 404, *p* = .02).

### 4.3 Generational transmission

#### 4.3.1 Inherited influences on offspring birth weight: maternal influences

Table 2 illustrates the unadjusted mother–offspring correlations separately for the conception groups. These correlations suggest the role of maternally provided genetic influences as the mother–offspring correlations are larger and significant for both the homologous (*r* mother height = .174; *r* mother weight = .290) and sperm donation groups (*r* mother height = .360; *r* mother weight = .204) than for the egg donation group (*r* mother height = .177; *r* mother weight = .023). The squared difference in the correlation coefficients for the combined homologous and sperm donation groups (*r* = .242 maternal weight; *r* = .216 maternal height) and the egg donation group gives an estimate of the proportion of variance attributable to maternally provided genetic effects (Table 1). For maternal weight, approximately 5% of the covariation with infant birth weight is attributable to maternal genetic effects (.242–.023)^2^, whereas for maternal height this figure is much smaller (0.15%) (.216–.174)^2^.

#### 4.3.2 Prenatal influences on offspring birth weight

The correlation between mother weight–offspring birth weight in the egg donation group is near zero (*r* = .023, *p* = .888) suggesting no intrauterine influences. However, although, the correlation is only approaching significance, it is in the expected direction and much larger for mother height–offspring birth weight (*r* = .177, *p* = .094) suggesting that intrauterine contributions may differ according to the measure of maternal stature used. The correlation in the egg donation group can only be due to intrauterine influences and suggests that approximately 3% of the covariance in birth weight and maternal height is due to intrauterine influences (.177^2^). Table 3 illustrates maternal and paternal correlations with infant birth weight. Greater similarity between mothers and offspring than between fathers and offspring is also indicative of prenatal influences on size at birth: within the homologous group where both parents are genetically related to the offspring, the association between parent height and birth weight is greater for mothers (*r* = .174, *p* = .006) than for fathers (*r* = .073, *p* = .376) which again, suggests intrauterine influences indexed by height but not weight.

#### 4.3.3 Inherited influences on offspring birth weight: paternal influences

The pattern of correlations in the sperm donation group suggests that paternal genetic factors are also important in the aetiology of birth weight in that the father–offspring correlations are smaller and non significant for the sperm donation group (*r* father height = .136, *p* = .373; *r* father weight = .036, *p* = .819) compared to the father–offspring correlations for the homologous and egg donation groups (where fathers pass genes on to their children). The only exception is for paternal height and infant birth weight where the correlation is small and non significant in both the homologous group where the father passes on genes and in the sperm donation group where the father does not. In general however, the unadjusted correlations appear to vary primarily according to whether the father is genetically related to the child. The square of the average correlation for the combined homologous and sperm donation group gives an estimate of the proportion of covariation with infant birth weight attributable to paternal genetic effects. For paternal height, this is 1% (.121^2^) and for paternal weight this is 5% (.218^2^).

### 4.4 Parent of origin inherited effects

Table 3 illustrates associations with offspring birth weight for maternal and paternal current weight and height. In terms of the relative magnitude of paternal and maternal genetic contributions, when both parents pass on genes to their offspring, there is evidence of a greater maternal than paternal genetic contribution (mothers in homologous group *r* = .260; fathers in homologous group *r* = .174). However, this greater maternal genetic contribution is not observed when only one parent in a dyad passes genes on to the child; the magnitude of association for (related) mothers in the sperm donation group (*r* = .196) and (related) fathers in the egg donation group (*r* = .274) is similar and in fact slightly higher in the egg donation fathers. Thus, the results suggest genetic contributions from both mothers and fathers. The only exception concerns the egg donation group where the father and not the mother are related. There, the (related) paternal contribution is significant and larger than the paternal contribution in the (related) homologous group (see Tables 2 and 3).

### 4.5 Adjusted parent–offspring associations

We next examined the pattern of mother–child and father–child association when controlling for the effect of potential confounding variables. The overall pattern of results was not altered when adjusting for the influences of child gender and family income (see Table 4). We also examined the influence of one maternally reported pregnancy complication because it was associated with lower birth weight at the level of a trend and because its frequency differed between the conception groups. Adjusting for hypertension in pregnancy in addition to child gender and family income again (Table 5) did not substantially alter the overall pattern of results. Overall, it clarified the pattern of results for maternal height and offspring birth weight in that the association between maternal height and offspring birth weight was increased in the egg donation group where mothers were not related to their child (*b* = 1871, *β* = .244, *p* = .025). That result thus highlights that intrauterine influences play a role in the association between maternal height and offspring birth weight. Height was measured in metres thus a 10 cm increase in maternal height is equivalent to an 18.7 g increase in infant birth weight (1871 × .1). Since the mother and offspring share no genes in common, this significant association must be attributed to the intrauterine environment provided by the mother.

### 4.6 Birth weight corrected for gestational age

Since birth weight is both a function of foetal growth and gestational age, we examined whether the pattern of correlation differed when birth weight corrected for gestational age was an outcome variable in this sample of singletons born at term (Table 6). In general, the pattern remained similar with significant correlations for the genetically related member of a dyad. Correlations were significant for mothers in the homologous group and for fathers in the egg donation group. However, the correlation of maternal height in the egg donation group was no longer significant illustrating that maternal height in part affects infant birth weight through influences on gestational age in addition to effects on foetal growth.

### 4.7 Examining genetic and intrauterine contributions to infant head circumference

Information on infant head circumference was available for 239 cases from antenatal records (most other infant growth measurements were not systematically recorded in antenatal records). Head circumference showed a positive correlation between maternal height in the related groups (combined homologous and sperm donation; *r* = .185, *p* = .01, *n* = 192) as well as the unrelated group (egg donation; *r* = .339, *p* = .021, *n* = 46). This pattern of results therefore suggested an intrauterine contribution to the relationship between maternal height and head circumference. Again, the pattern of results was different for maternal weight and there was evidence of a smaller but largely genetic contribution. Maternal weight at antenatal booking and head circumference were correlated in the related groups (*r* = .172, *p* = .032, *n* = 156) but not the unrelated group (*r* = −.103, *p* = .558, *n* = 35).

## 5 Discussion

Using this novel design, we found a different pattern of results depending on the measure of parental size examined. The contribution of parental weight to offspring birth weight was explained by genetic factors whereas intrauterine influences were apparent when maternal height was examined as a predictor variable. For parental weight, in both unadjusted correlations and adjusted multiple regression analysis, significant parent weight–offspring birth weight associations were only seen when the parent was genetically related to the child. Both maternal and paternal genetic factors made a contribution. Moreover, results were similar when using two assessments of maternal weight; one objective measure assessed at the time of pregnancy and one self report measure assessed some years after the birth of the child. In families where both parents passed genes on to the child (the homologous IVF group), there was evidence of a greater maternal than paternal genetic contribution to infant birth weight as has been observed in previous studies using standard family data [23,34]. Moreover, within this group, the magnitude of the association between parental weight (*r* = .260 mothers; *r* = .174 fathers) and offspring birth weight was in fact very similar to a large population-based study of the familial transmission of birth weight (*r* = .254 mothers; *r* = .161 fathers) [20].

One interesting observation was that the greater maternal genetic contribution that was seen when both parents were genetically related to the child was not observed when only a single parent in a pair passed genes on to the child: in fact mothers in the sperm donation group and fathers in the egg donation group showed very similar sized correlations with offspring birth weight. This raises the possibility of biological interactions between maternal and paternal genetic contributions. In fact, the contribution of paternal genetic factors to birth weight differed depending on whether the mother also contributed her genes. When examining paternal height, there was a difference in the magnitude of the contribution of paternal factors in the egg donation and the homologous groups. In both these groups, the father is genetically related to the child, although the correlation was significant only in the egg donation group. One interpretation of this finding is that competition between maternal and paternal genes could be influencing foetal growth [35,36]. In evolutionary terms, it is in the father's interests to have a large, robust child likely to survive to reproductive age and pass paternal genes onto the next generation. In contrast, it is in the mother's interest to constrain the growth of the infant to some degree as she must transfer nutrients to the offspring and the greater the nutritional demands of a pregnancy, the greater the cost to the mother's potential future reproductive fitness. The fact that we observe a larger paternal contribution in the egg donation group suggests the possibility of a biological interaction between the intrauterine environment and maternal inherited characteristics which suppresses the father's genetic influence. Only in the egg donation group is there a separation of maternal inherited characteristics and the intrauterine environment and it is here that the effect of paternal genes is greatest. We are only able to postulate on what this mechanism may be but it could perhaps involve the action of imprinted genes in the placenta (i.e. differential expression of a gene dependent on which parent it is inherited from) [35,36].

The results that examined the association between birth weight and parent height—a measure of adult stature independent of obesity showed evidence of a contribution of the intrauterine environment in that significant mother–offspring correlation was observed in the egg donation group despite the fact that mother is not genetically related to the child (although she did experience the pregnancy and provide the intrauterine environment). The association between maternal height and infant birth weight in the egg donation group is therefore attributable to intrauterine effects that are independent of the link between the foetal and maternal genomes. Although the numbers were small, a similar pattern of results was observed for head circumference. Taken together, this pattern of results suggests that maternal height is a more accurate estimate of the constraint that maternal size exerts on the growth of the foetus as opposed to maternal weight. These results are in line with a previous study that examined the contributions of egg donor and egg recipient height and weight to offspring birth weight [24]. That study found evidence of both genetic and intrauterine influences on offspring birth weight when recipient and donor weight was examined but only intrauterine influences when height was examined. These results in combination suggest that maternal height provides a better index of intrauterine influences on offspring birth weight than maternal weight.

The results of the analysis of birth weight corrected for gestational age illustrated that maternal height influenced length of gestation. When examining the association of maternal height with infant birth weight corrected for gestational age, the correlation did not reach significance in the egg donation group. Although we restricted our analysis to singletons born at term, birth weight is an index of both foetal growth and gestational age [25]. The fact that the index of intrauterine influences (the maternal–offspring correlation in the egg donation group) did not reach significance when examining birth weight adjusted for gestational age illustrates that part of the effect of maternal height on birth weight is due to intrauterine influences on both gestational age and foetal growth.

The research design employed included children conceived via IVF and allowed the comparison of paternal and maternally provided genetic influences as well as an examination of the contribution of the intrauterine environment independent of the link between the foetal and maternal genomes. However, the focus on offspring born following IVF means that there may be some concern about the representativeness of the sample and the generalizability of results. Studies have consistently shown that children conceived via assisted reproductive technologies do not differ from children conceived naturally in terms of psychological adjustment [37] and a previous report on this sample similarly reports no differences [32]. The sample on which this report is based is also comparable to population-based data in terms of parent mental health and family income although it is not representative in terms of antenatal risk [27,32]. The sample includes families from a range of demographic backgrounds in part because IVF treatment is freely provided by the National Health Service in the UK given certain eligibility criteria. Although some studies have reported that birth weight is reduced in children conceived via assisted reproduction [38], we found that although the average birth weight of infants in this sample was slightly lower that with UK population-based data the absolute difference was very low (8 g on average) [33]. Despite increased rates of certain antenatal risks in this sample (e.g. high blood pressure/oedema) compared to naturally conceived pregnancies, all comparisons of association were conducted between mothers who conceived via IVF, therefore, the three conception groups were similar in terms of method of conception and having a history of fertility problems. We found no association between parity and birth weight despite associations in general population studies [25]. This seems likely due to the high proportion of primiparous women in our sample. There have been some reports of increased rates of imprinting related disorders in children conceived via assisted reproductive technologies [39]. However, given the very low prevalence of these disorders in the general population, these claims have not yet been substantiated [40] and no children in the present sample were described as having imprinting disorders. We only had information on parental weight available and were not able to examine the generational transmission of birth weight which it has been reported shows transmission through the maternal rather than paternal line [41]. Self-reported parental height and weight measurements are also a limitation although we were able to validate maternal reports against information in the antenatal records to show that maternal self reports were reliable and valid. However, we did not have validation information available for fathers. Men have been shown to be slightly less reliable than women in reporting their height [42,43] although in general, the absolute differences between reported and measured height are small for both genders. Nevertheless, the magnitude of the correlations with birth weight that we observed was very similar to those observed in a UK family study of naturally conceived children that used measurements of parental size taken by trained midwives [22]. Finally, for a small proportion of mothers who gave consent to review antenatal records, the records could not be traced. Assortative mating for parental height and weight may also be an issue although this would only affect the relative genetic contribution from parents to offspring in the homologous group where both parents are related to the child. In the homologous group, parent current weights were correlated (*r* = .261, *p* = .002). This association could be due to either assortative mating or to shared environmental contributions. However, since there was no evidence of a significant correlation in parental height (*r* = .034, *p* = .663), this suggests that the parent correlation in weight is due to shared environmental factors. A final limitation is that we cannot exclude the possibility of clinics matching gamete donors and recipients on height. However, this seems unlikely to account for our results for a number of reasons. Firstly, because we excluded related donors and secondly, because given the limited choice of donors available in the UK, close matching of physical characteristics between recipient and donor is impossible [44]. Furthermore, recent UK guidelines suggest that close matching is not necessarily desirable [44].

## 6 Conclusion

In summary, we find that genetic factors explain the association between parental weight and infant birth weight but that there is a contribution of the intrauterine environment on the association between maternal height, infant birth weight and head circumference. Presumably maternal stature as measured by height puts a physical constraint on the growth of the foetus. The intrauterine influence of maternal height on birth weight appears to work both through influences on foetal growth and on gestational age. There was also evidence that could be interpreted as suggesting a biological interaction between maternal inherited characteristics and the intrauterine environment that suppresses the contribution of paternal genes to birth weight.

## Figures and Tables

**Table 1:** Maternal and paternal factors contributing to mother/father and offspring similarity in the three conception groups.

Conception group	Mother contributions to offspring birth weight	Father contributions to offspring birth weight
Homologous	Gm + U	Gf
Sperm donation	Gm + U	–
Egg donation	U	Gf

Gm = maternally provided genes. Gf = paternally provided genes. U = intrauterine environment.

**Table 2:** Mother–offspring correlations for each conception group.

	Mother	*N*
Homologous: (Mother genetically related)		
Weight at antenatal booking	*r* = .290***	134
Height	*r* = .174**	249
Sperm donation: (Mother genetically related)		
Weight at antenatal booking	*r* = .204	41
Height	*r* = .360**	65
Egg donation: (Mother genetically unrelated)		
Weight at antenatal booking	*r* = .023	40
Height	*r* = .177^a^	91

^a^*p* < .1. *p < .05. **p < .01. ***p < .001. *r* = Pearson's correlation. In all conception groups the mother experiences the pregnancy and provides the intrauterine environment.

**Table 3:** Comparing maternal and paternal genetic contributions to offspring birth weight.

	Mother			Father		
	*r*	*p*	*n*	*r*	*p*	*n*
*Homologous (mother and father genetically related)*						
Height	.174	.006	249	.073	.341	170
Current weight	.260	.001	237	.174	.029	155

*Sperm donation (mother only genetically related)*						
Height	.360	.003	65	.136	.373	45
Current weight	.196	.133	60	.036	.819	43

*Egg donation (father only genetically related)*						
Height	.177	.094	91	.274	.041	56
Current weight	−.021	.839	93	.297	.027	55

*r* = Pearson's correlation. *n* = sample size for the analysis.

**Table 4:** Mother–offspring and father–offspring associations adjusted for demographic factors.

	Mother			Father		
	*b*	*β*	*p*	*b*	*β*	*p*
*Homologous (mother and father genetically related)*						
Maternal pregnancy weight	13.17	.286	.001	–	–	–
Current weight	11.09	.266	.001	7.10	.181	.029
Height	1403.35	.184	.004	683.28	.090	.248

*Sperm donation (mother only genetically related)*						
Maternal pregnancy weight	9.68	.247	.130	–	–	–
Current weight	10.90	.278	.033	1.43	.032	.840
Height	3213.07	.432	.001	628.01	.096	.540

*Egg donation (father only genetically related)*						
Maternal pregnancy weight	1.25	.024	.890	–	–	–
Current weight	–0.73	−.017	.878	9.31	.309	.024
Height	1491.32	.194	.080	2263.66	.281	.041

Coefficients adjusted for child gender and family income. *b* = unadjusted beta coefficient and shows the unit (kilogram/metre) increase in parental weight/height per unit increase (grams) in birth weight. *β* = standardized beta coefficient (which can be interpreted in the same way as a correlation coefficient).

**Table 5:** Mother–offspring and father–offspring associations adjusted for demographic and antenatal factors.

	Mother			Father		
	*b*	*β*	*p*	*b*	*β*	*p*
*Homologous (mother and father genetically related)*						
Maternal pregnancy weight	13.36	.290	.001	–	–	–
Current weight	11.27	.270	.001	6.34	.161	.057
Height	1407.51	.184	.004	653.59	.087	.272

*Sperm donation (mother only genetically related)*						
Maternal pregnancy weight	8.96	.228	.169	–	–	–
Current weight	10.50	.268	.045	1.24	.028	.862
Height	3197.22	.430	.001	542.54	.083	.607

*Egg donation (father only genetically related)*						
Maternal pregnancy weight	2.81	.054	.762	–	–	–
Current weight	2.31	.053	.620	10.94	.364	.007
Height	1871.23	.244	.025	2080.64	.258	.057

Coefficients adjusted for child gender, family income and high blood pressure in pregnancy. *b* = unadjusted beta coefficient and shows the unit (kilogram/metre) increase in parental weight/height per unit increase (grams) in birth weight. *β* = standardized beta coefficient.

**Table 6:** Mother–offspring and father–offspring associations with birth weight adjusted for gestational age.

	Mother			Father		
	*r*	*p*	*n*	*r*	*p*	*n*
*Homologous (mother and father genetically related)*						
Maternal pregnancy weight	.292	.001	134	–	–	–
Current weight	.242	.001	237	.142	.079	155
Height	.165	.009	249	.051	.500	174

*Sperm donation (mother only genetically related)*						
Maternal pregnancy weight	.100	.535	41	–	–	–
Current weight	.278	.033	60	–.072	.648	43
Height	.201	.108	65	.099	.517	45

*Egg donation (father only genetically related)*						
Maternal pregnancy weight	.011	.944	40	–	–	–
Current weight	.026	.805	93	.310	.021	55
Height	.102	.334	91	.306	.022	56

Coefficients adjusted for gestational age. *r* = Pearson's correlation. *n* = sample size for the analysis.
